# Efficient imaging and computer vision detection of two cell shapes in young cotton fibers

**DOI:** 10.1002/aps3.11503

**Published:** 2022-11-26

**Authors:** Benjamin P. Graham, Jeremy Park, Grant T. Billings, Amanda M. Hulse‐Kemp, Candace H. Haigler, Edgar Lobaton

**Affiliations:** ^1^ Department of Crop and Soil Sciences North Carolina State University Raleigh North Carolina 27695‐7620 USA; ^2^ Department of Plant and Microbial Biology North Carolina State University Raleigh North Carolina 27695‐7612 USA; ^3^ Department of Computer Science North Carolina State University Raleigh North Carolina 27695‐8206 USA; ^4^ Bioinformatics Graduate Program North Carolina State University Raleigh North Carolina 27695‐7566 USA; ^5^ Genomics and Bioinformatics Research Unit, U.S. Department of Agriculture, Agricultural Research Service Raleigh North Carolina 27606‐7825 USA; ^6^ Department of Electrical and Computer Engineering North Carolina State University Raleigh North Carolina 27695‐7911 USA

**Keywords:** cotton diversity, fiber morphogenesis, *Gossypium barbadense*, *Gossypium hirsutum*, light microscopy, machine learning

## Abstract

**Premise:**

The shape of young cotton (*Gossypium*) fibers varies within and between commercial cotton species, as revealed by previous detailed analyses of one cultivar of *G. hirsutum* and one of *G. barbadense*. Both narrow and wide fibers exist in *G. hirsutum* cv. Deltapine 90, which may impact the quality of our most abundant renewable textile material. More efficient cellular phenotyping methods are needed to empower future research efforts.

**Methods:**

We developed semi‐automated imaging methods for young cotton fibers and a novel machine learning algorithm for the rapid detection of tapered (narrow) or hemisphere (wide) fibers in homogeneous or mixed populations.

**Results:**

The new methods were accurate for diverse accessions of *G. hirsutum* and *G. barbadense* and at least eight times more efficient than manual methods. Narrow fibers dominated in the three *G. barbadense* accessions analyzed, whereas the three *G. hirsutum* accessions showed a mixture of tapered and hemisphere fibers in varying proportions.

**Discussion:**

The use or adaptation of these improved methods will facilitate experiments with higher throughput to understand the biological factors controlling the variable shapes of young cotton fibers or other elongating single cells. This research also enables the exploration of links between early cell shape and mature cotton fiber quality in diverse field‐grown cotton accessions.

Computer vision is a field of artificial intelligence that enables automated data extraction from digital images, including techniques such as object detection and classification as will be described here. Recently, computer vision has been increasingly used in agriculture to enhance crop research, production, and breeding (reviewed by Kakani et al., 2020; Paul et al., 2020; Tian et al., 2020; Li et al., 2020b). Examples of effective implementations of computer vision include monitoring crop development (Sadeghi‐Tehran et al., 2017), identifying plant diseases (Toseef and Khan, 2018) and pests (Maharlooei et al., 2017), and increasing the efficiency of irrigation (Nhamo et al., 2018). Specifically for cotton (*Gossypium* L.), computer vision has been used to: diagnose disease (Toseef and Khan, 2018); detect boll maturity (Li et al., 2020a); and map cotton boll number, volume, and distribution in the field (Sun et al., 2018, 2020). Here, we demonstrate a less common use of computer vision for cellular phenotyping—specifically, for the annotation of two cell shapes that may occur in young cotton fibers.

Cotton fibers are highly elongated and thickened extensions of the seed epidermis that provide a key renewable resource for textile manufacture (Fang, 2018; Kim, 2018). Two allotetraploid species, *G. hirsutum* L. (*Gh*) and *G. barbadense* L. (*Gb*), produce most of the worldwide fiber crop (about 90% produced by *Gh*, 5% by *Gb*, and the remainder by a few diploids in minor cultivation) (Constable et al., 2015). Although *Gh* is more productive, its fiber quality is lower than that of *Gb*. Researchers are actively seeking insights into the mechanisms underlying the superior length, strength, and fineness of *Gb* cotton (for examples, see Tuttle et al., 2015; Brown et al., 2020; Wang et al., 2020; Pei et al., 2021; Yu et al., 2021; Zhao et al., 2021). However, definitively linking the higher quality of *Gb* fiber to particular cellular or genetic mechanisms is yet to be accomplished.

We previously studied an elite cultivar (cv.) of each species, *Gb* cv. Phytogen 800 and *Gh* cv. Deltapine 90, to understand the control of fiber diameter (Stiff and Haigler, 2016; Pierce et al., 2019; Graham and Haigler, 2021). The diameter of the mature fiber cell contributes to fiber fineness (or mass per unit length) in tandem with the extent of secondary wall thickening. *Gb* cotton is composed of smaller‐diameter mature fibers that are preferred for spinning the fine, strong yarns used to manufacture the highest‐quality cotton fabrics (Kelly et al., 2015). The suboptimal fineness of *Gh* fiber (e.g., in Deltapine 90 versus Phytogen 800; Avci et al., 2013) may relate to *Gh* producing both wide fibers (called hemisphere) and narrow fibers (called tapered), whereas *Gb* produces fibers with one narrow shape that appear similar to *Gh* tapered fibers (Figure 1). These differences between fiber shapes are apparent at the fiber apex on the first day after fiber initiation and extend down the shank as the fiber elongates. The narrow and wide *Gh* fiber shapes stabilize by the second day after anthesis and persist through fiber maturity (Pierce et al., 2019; Graham and Haigler, 2021).

**Figure 1:**
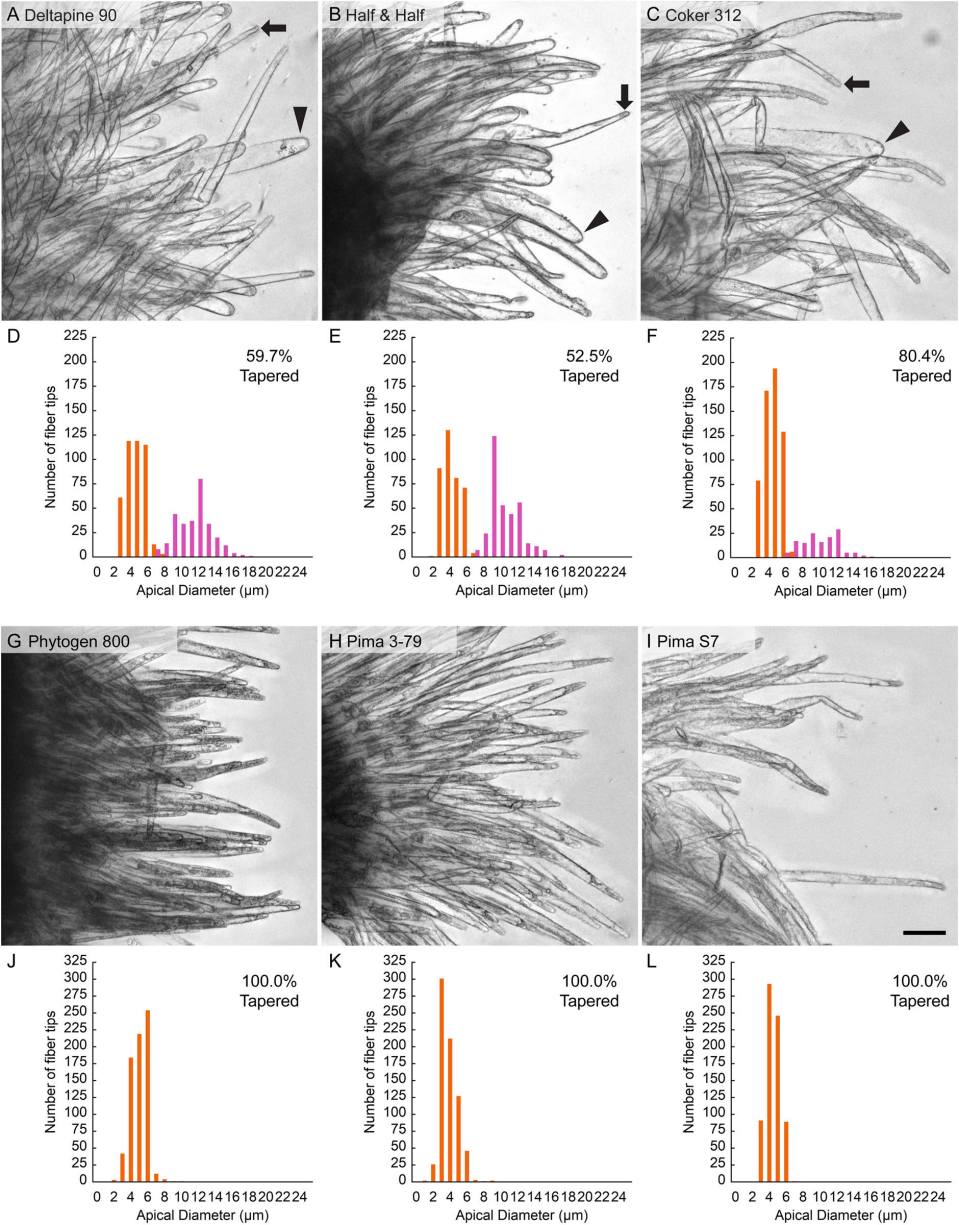
Representative images and manually annotated distributions of apical diameter for six cotton accessions tested by computer vision. (A–C) In images of three *Gossypium hirsutum* (*Gh*) accessions (A, Deltapine 90; B, Half and Half; C, Coker 312), tapered or hemisphere fibers are indicated by an arrow or arrowhead, respectively. (D–F) Bimodal apical diameter distributions for the *Gh* accessions pictured in A–C, with tapered or hemisphere shapes in orange or magenta, respectively. (G–I) Images of three *G. barbadense* (*Gb*) accessions (G, Phytogen 800; H, Pima 3‐79; I, Pima S7) show only tapered fibers with a (J–L) unimodal distribution. A total of 720 fibers per accession were manually annotated, representing nine ovules from three bolls of different plants. The 50 µm scale bar in panel I applies to all images.

Different apical shapes, diameters, and other cellular characteristics in the tip region distinguish three fiber morphogenetic fates: two in Deltapine 90 versus one in Phytogen 800 (Stiff and Haigler, 2016; Pierce et al., 2019; Graham and Haigler, 2021). In theory, the tapered fibers in *Gh* cotton would be a positive contributor to optimal fiber fineness, making it necessary to examine more cotton accessions to determine whether the results from the two originally studied cultivars are typical of the species and whether variability in early cotton fiber morphology impacts mature fiber quality. However, the currently used manual methods for determining the proportion of narrow versus wide fibers within a population are laborious, requiring researchers to image the fibers of one sample in multiple focal planes, generate a stacked image for each field of view, and annotate fiber shapes by hand (Stiff and Haigler, 2016; Pierce et al., 2019; Graham and Haigler, 2021). It was not practical to extend these manual methods to analyzing the shape of young fibers in numerous cotton accessions, which is a key next step in this research area as we work toward understanding the biological controls of this trait and the potential relationship to the higher quality of *Gb* versus *Gh* cotton.

Here, we describe newly developed, semi‐automated, efficient protocols for imaging and annotating the shape of young cotton fibers using computer vision. For the benefit of diverse readers, an overview of the problem addressed and the machine learning (ML) approaches that we developed and employed are shown in Figure 2. The results show that object detection within extended depth of field images can automatically and reliably characterize cotton fibers as having tapered or hemisphere shapes. The effectiveness and robustness of computer vision is demonstrated by the similarity of outcomes to fiber shape annotations performed manually for three *Gh* accessions (Deltapine 90, Half and Half, and Coker 312) and three *Gb* accessions (Phytogen 800, Pima 3‐79, and Pima S7). All three *Gb* accessions had nearly 100% tapered fibers, whereas the percentage of tapered versus hemisphere fibers varied between *Gh* accessions. These improved methods expand our ability to: carry out experiments on the molecular control of this trait; phenotype young fiber morphology in numerous cotton accessions, including those grown in the field; and explore linkages between early fiber shape and mature cotton fiber quality.

**Figure 2:**
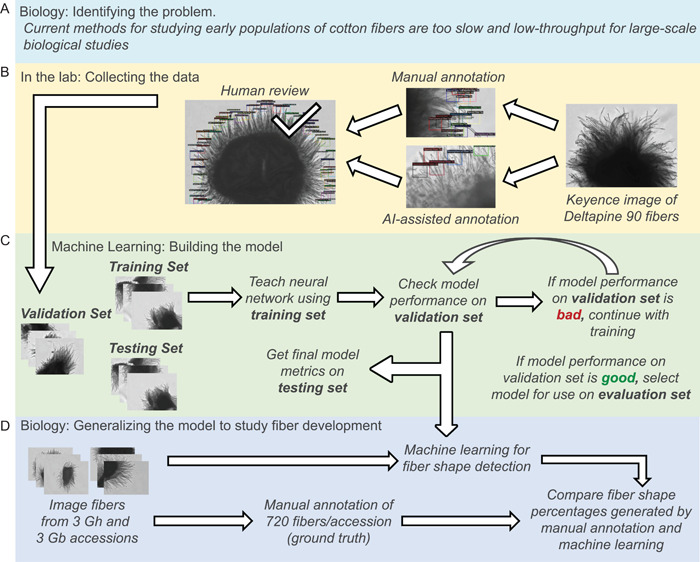
Schematic diagram of the research need (A), methods (B, C), and results (D). In the machine learning field, the ground truth is established by a manual evaluation of the same samples as a means of assessing the reliability of the machine learning algorithm. AI, artificial intelligence.

## METHODS

### Plant materials

The cotton plants were grown in a greenhouse with a 26/22°C diurnal cycle in the North Carolina State Phytotron, as described previously (Pierce et al., 2019). Flowers were tagged on the day of anthesis and harvested at three days post anthesis (3 DPA). Although the distinct fiber shapes had already formed by 2 DPA (Stiff and Haigler, 2016; Graham and Haigler, 2021), we chose to image at 3 DPA for two reasons. First, the fibers were still relatively short, which minimized entanglement and facilitated the visualization of multiple fiber tips in each image. Second, imaging at 3 DPA helped to address the theoretical possibility that fiber tapering in some accessions might be delayed beyond 1 to 1.5 DPA as observed for *Gh* cv. Deltapine 90 or *Gb* cv. Phytogen 800, respectively (Graham and Haigler, 2021).

### Cotton fiber sample preparation, digital image collection, and image analysis

Ovules with attached fiber were fixed in the greenhouse. The fixative previously used (HistoChoice, formerly available from Amresco) (Stiff and Haigler, 2016; Pierce et al., 2019; Graham and Haigler, 2021) is obsolete, which led to the testing and validation of another low‐toxicity, formalin‐free fixative (A5472; Sigma‐Aldrich, St. Louis, Missouri, USA; Appendix S1). To remove the boll wall and harvest undamaged tissue, shallow longitudinal incisions were made between the locule walls, and lateral incisions were made 3 mm from the top and around the base of the boll. The ovules with attached fibers were lifted out of all of the locules of each boll and fixed (1 h at ambient temperature, approximately 1:10 v/v tissue:fixative) prior to optional storage at 4°C. Immediately before imaging, the ovules were examined under a stereomicroscope (incident light, black background, 31×) and three vigorous ovules were selected from each boll, taking care that the ovules did not dry out. The ovules were rinsed three times (5 min each) in buffer (0.05 M PIPES, 12 mM EGTA, 5 mM EDTA, and 0.1% [w/v] Tween 80, pH 6.8), which had a lower osmolarity than the microtubule‐stabilizing buffer previously used for aldehyde‐fixed fibers (Seagull, 1990; Graham and Haigler, 2021). While steadying an ovule with forceps, one to three small pieces of its chalazal end with attached fibers were dissected away using a small knife (#10055‐12; Fine Science Tools, Foster City, California, USA). Each ovule piece was placed in a single well of a 24‐well slide (#63430‐04; Electron Microscopy Sciences, Hatfield, Pennsylvania, USA) and mounted in buffer. A 24 × 60 mm coverslip was applied to the filled slide and sealed with petroleum jelly.

The samples were imaged using brightfield optics and default settings for the 2.83 megapixel color CCD camera of the Keyence BZ‐X810 imaging system (Keyence Corporation, Itaska, Illinois, USA) at the Cellular and Molecular Imaging Facility of North Carolina State University. The location of each sample was identified visually using a 2× objective and mapped using the navigation function of the integrated Keyence software. Using the 10× objective lens (plan‐apochromatic with 0.45 numerical aperture) and 60% closed condenser aperture setting, a region with many fiber apices was selected for imaging using the multi‐point and z‐stack capture functions. The precise location was recorded by the software prior to setting the limits of the z‐plane range visually (1.2 µm step size). Typically, three 24‐sample slides (representing three accessions) were set up in parallel prior to the automatic capture of TIFF images in this study. (Alternatively, JPEG images can be captured—see further details in the Discussion.) The captured z‐stacks for each sample were processed into a two‐dimensional image using the full‐focus function of the software.

Most of the first set of dissected Coker 312 samples (one of six accessions analyzed) had many particles adhering to the fiber surfaces. A second set of dissected samples was cleaner, implying that the original problem was an accidental outcome of dissection. Cleanliness of the samples should be assessed prior to continuing with the automated image capture.

The evaluation test sets were analyzed with our best‐performing ML algorithm and manually (to determine the ground truth for comparison with the computer vision results). In the manual process, a total of 720 fibers per accession were systematically chosen from 24 images in a way that minimized bias (see details in the section below, “Evaluation of diverse cotton accessions”). Each fiber was inspected by eye and annotated as tapered or hemisphere. The apical diameter was also measured as described previously (Stiff and Haigler, 2016; Pierce et al., 2019, Graham and Haigler, 2021) by determining the diameter of the smallest circle that would fit within the apical dome. The two fiber shapes were readily distinguished visually. Hemisphere fibers have a rounded apex with a clear lumen (due to the large central vacuole being near the apex) and a lesser diameter increase immediately below the apex. Tapered fibers have a pointed apex with dense cytoplasm (due to a remote central vacuole) and a greater diameter increase below the apex (Stiff and Haigler, 2016; Graham and Haigler, 2021). All annotated fibers were substantially longer than progenitor “pre‐taper” fibers, implying that they had passed through the phase of tip morphogenesis (Graham and Haigler, 2021); in general, the annotated fibers were at least 50% of the maximal length observed and relatively separated on the edge of the fiber mass (Figure 1).

### Computer vision

**Figure 3:**
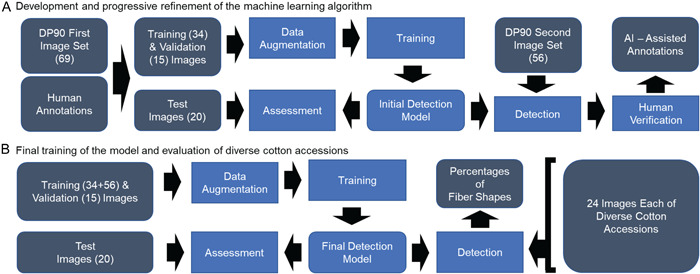
Workflows for the progressive training and use of the model. (A) Training the initial model including human (manual) annotation and artificial intelligence (AI)‐assisted annotation of images of 3 DPA fibers of *Gossypium hirsutum* (*Gh*) cv. Deltapine 90 (DP90). (B) Training the final model and using it to determine the percentage of the two fiber shapes in six cotton accessions.

This research proceeded in two major steps. First, we developed and progressively refined an ML algorithm, including the use of manual and artificial intelligence (AI)‐assisted annotation (Figure 3A). Second, we carried out the final training of the model and evaluated test sets representing six cotton accessions (Figure 3B). These processes are described in more detail below.

#### Object detection model

We used an object detection model for the identification of cotton fiber tips within the microscope images. An object detection pipeline uses images as input and returns bounding boxes indicating the location of instances and class type of the objects found. In the current case, the bounding boxes enclosed the cotton fiber tips and identified them as tapered or hemisphere (Figure 2). To train an object detection model, we required a training set of images with the corresponding annotations. Most state‐of‐the‐art models for object detection are currently based on deep learning architectures, which learn feature extraction from images and the detection task concurrently. Training an entire deep learning model requires a large number of samples: initial deep learning architectures such as AlexNet used millions of images, and current state‐of‐the‐art models such as Vision Transformers use billions of images (Krizhevsky et al., 2017; Zhai et al., 2021). Instead, we employed transfer learning, which makes use of a pre‐trained model for object detection by fixing the feature extraction portion of the network, while training only the detection task portion. This often requires hundreds to thousands of samples depending on the application.

The specific deep learning model used here was Detectron2 (facebook research/detectron 2, https://github.com/facebookresearch/detectron2), which is widely used, including in agricultural studies (da Silva et al., 2020). It relies on a backbone architecture, for which we chose Faster RCNN R50‐FPN 3x (https://github.com/facebookresearch/detectron2/blob/main/MODEL_ZOO.md) due to its good performance on the COCO Object Detection Baselines (https://cocodataset.org/#home).

#### Manual image annotation for training

The training data consisted of 125 unique images of 3 DPA *Gh* cv. Deltapine 90 fibers, which were derived from at least 48 ovules (eight ovules from each of six bolls harvested from independent plants). As shown in Figure 3A, we originally used 69 out of 125 images to train the fiber tip detection model. In the Labelbox platform (https://labelbox.com), one cotton expert and two ML graduate students annotated all (4466) of the visible fiber tips (see above for details). This resulted in an average of 64.7 annotations per image, with 63.9% of the fibers being labeled as tapered and 35.22% of the fibers being labeled as hemisphere; a small number of fibers (0.87%) were labeled as undecided and not reviewed further. This manual labeling required about 8.8 min/image, which would likely be reduced with more experience for all labelers. The Labelbox data set generated the bounding box annotations used to train the object detection model. The first version of the model did not perform as desired, prompting next steps as described below.

#### AI‐assisted image annotation for training

In order to improve model performance, we used an AI‐assisted annotation of fibers in the remaining 56 images of the training data, which required less time than the manual process (see Figure 3A). We used the best model available at that time that had been trained on the original set of manually labeled images to predict initial bounding boxes for the 56 new images. This was followed by human verification of accurate labeling in Roboflow (https://roboflow.com), which required about 2.9 min/image including slight adjustments of labels in a few instances, compared to 8.8 min/image for fully manual labeling. Among the 2029 predictions within the 56 images (36.2 predictions/image), there were 57.7% tapered fibers and 42.3% hemisphere fibers. After human verification, 1969 annotations remained (35.2 annotations/image) with 60.7% tapered fibers and 39.3% hemisphere fibers. Although the AI‐assisted process resulted in fewer annotations (35.2 labels/image) than the fully manual method (64.7 labels/image), the addition of AI‐assisted labels was an efficient way to improve model performance (see below).

We partitioned the 69‐image manual annotation data set into a 34‐15‐20 image train‐validation‐test split (49‐22‐29%, respectively). The training and validation sets were split randomly using Roboflow, and the test set of images from a separate collection day was selected manually to test the ability to generalize the results using new image sets. We added all of the 56 AI‐assisted images to the training set (resulting in a 72‐12‐16% train‐validation‐test split), allowing us to demonstrate the performance benefits on the same test set arising from our AI‐assisted labeling method (see Table 1). We prepared the data sets in Roboflow, which allowed us to split the data and add image preprocessing and augmentation, including auto‐orient, grayscale, horizontal and vertical flips, and 180‐degree rotations. Roboflow uses these augmentations to create new training examples, allowing us to multiply the training set up to three times for better performance. This data augmentation process (Figure 3B) resulted in a final training set composed of 237 images, with 6464 (64.5%) and 3554 (35.5%) instances of tapered and hemisphere fibers, respectively.

**Table 1:** Comparison of the performance of the machine learning (ML) model after training on only manual (M) labels or a combination of manual and AI‐assisted (M + AI) labels. The same 15 validation (V) images and 20 test images of *Gossypium hirsutum* (*Gh*) cv. Deltapine 90 were used throughout.

Input labels	Image sets	AP50	AP for H fibers	AP for T fibers	M % T	ML % T	Difference %T, M vs. ML	M fiber number	ML fiber number
M	15 V	44.99	41.33	28.59	60.0	47.0	13.0	851	455
M	20 test	38.33	35.70	23.80	63.5	48.9	14.6	1687	687
M + AI	15 V	48.38	30.05	44.40	60.0	68.8	8.8	851	552
M + AI	20 test	42.48	27.56	36.97	63.5	68.8	5.3	1687	814

*Note*: AP = average precision; H = hemisphere fiber; T = tapered fiber.

#### Model training and assessment

The models were trained and evaluated in Google Colab on their free GPUs (https://colab.research.google.com/). For the model training, we initialized the model with the default weights and parameters of Faster RCNN R50‐FPN 3x from Detectron2, which is pretrained on COCO data sets. We trained the model on the full image size for 1000 iterations, evaluating the model every 100 iterations on the validation set and saving each model (Appendix S2). Because we performed the data augmentation in Roboflow, we turned off the default data augmentation in Detectron2. Hyperparameter settings are available at the project GitHub (see Data Availability statement).

We attempted to address the class imbalance in the training set (64.5% tapered and 35.5% hemisphere) using various data augmentation techniques, such as including images with a majority percentage of hemisphere fibers, but none improved model performance. In addition, we added existing annotations and images collected with another imaging system (Olympus BH‐2 microscope, Q‐Color 5 megapixel camera; Olympus/Evident, Tokyo, Japan) to the initial training set, but training on Keyence images alone gave the best results when Keyence images were the evaluation target. Those who have a different imaging system may consider training a new model on their data sets.

We were seeking a final model that performed well on the validation and test sets and that would also generalize well to the evaluation sets of six cotton accessions. One criterion for model selection was the AP50 value, which is derived from the average precision (AP), another standard metric in object‐detection tasks (https://cocodataset.org/#detection-eval). To further define AP50, given a ground truth bounding box and a predicted bounding box, the intersection over union (IoU) value is defined as the area of their intersection divided by the area of their union. In the case of AP50, if a predicted bounding box exceeds 50% for IoU with a ground truth bounding box, then it is a “good” prediction, also known as a true positive. Thus, the AP50 will increase with greater IoU between the ground truth bounding boxes and the predicted bounding boxes.

We determined the best‐performing model by selecting the model with the highest AP50 value on the 15‐image validation set. We then observed the model performance on the 20‐image test set of unseen Deltapine 90 images. The model was first trained on manually labeled images and then on images that had both manual and AI‐assisted labels, with the latter improving the performance of the model on the validation and test image sets (Table 1). Specifically, the AP50 values increased by 3.4 or 4.1 units when AI‐assisted labels were added to the validation or test sets, respectively. Adding AI‐assisted labels also brought the ML prediction of the percentage of tapered fibers closer to the ground truth. For the validation set, a difference of 13.0% between the ML predictions and manual labels was reduced to 8.8% using AI‐assisted labeling. In the test set, the difference was reduced from 14.6% to 5.3% when implementing AI‐assisted labeling (Table 1). In summary, AI‐assisted labeling allowed us to improve model performance by adding more labels in less time than is required for manual labeling.

#### Evaluation of diverse cotton accessions

The model was further evaluated using images from three *Gh* accessions (Deltapine 90, Half and Half, and Coker 312) and three *Gb* accessions (Phytogen 800, Pima 3‐79, and Pima S7). The 24 images of each accession derived from nine ovules (three ovules from each of three bolls harvested from independent plants) and are available online (see Data Availability statement). The Deltapine 90 images were a separate collection to those used for training. To generate manual annotations (ground truth) for each accession in minimal time while minimizing bias, 30 long fibers were labeled as tapered or hemisphere while systematically working left‐to‐right in each of 24 images per accession while avoiding out‐of‐focus tips. The percentage of tapered fibers among the 720 hand‐annotated fibers per accession was calculated for comparison with the results generated by the computer vision algorithm for the same images.

#### Statistical evaluation

A Bayesian statistical analysis was performed to compare the effects of the annotation method and number of images on the mean and precision of the tapered fiber percentages. The tidyverse package (Wickham et al., 2019) in R (R Core Team, 2022) was used for data organization and visualization. The model was fitted in the brms package (Bürkner, 2017, 2018) in R, an interface to Stan (Carpenter et al., 2017; Stan Development Team, 2022), using uninformative priors assuming a binomial likelihood for the posterior distribution. Our initial analysis showed that the data were overdispersed compared to the binomial distribution, so a group‐level (random) image effect, allowing for heteroskedasticity between the annotation methods, was included in the final model to capture added variance. The emmeans (Lenth, 2022) and tidybayes (Gabry et al., 2019; Gabry and Mahr, 2022) packages were used to plot median point estimates and 66% and 95% credible intervals from the posterior distribution. The code is available at the project GitHub (see Data Availability statement).

## RESULTS

Using the trained computer vision model, we evaluated images from six cotton accessions, three for *Gh* (Figure 1A–C) and three for *Gb* (Figure 1G–I), for which the ground truth had been established by manually annotating 720 fibers within 24 images per accession. In addition, the apical diameters of all fibers in the evaluation sets were measured by hand (Figure 1D–F, J–L; Appendix S3). The results for *Gh* cv. Deltapine 90 and *Gb* cv. Phytogen 800 were similar to prior results (Appendix S3; Pierce et al., 2019; Graham and Haigler, 2021), and the results for the four additional accessions broadened the species‐specific patterns. The apical diameters of the 3 DPA fibers had a bimodal distribution for all three *Gh* accessions, but the percentage of tapered fibers was much greater for Coker 312 than for Deltapine 90 and Half and Half (Figure 1D–F). All three *Gb* accessions showed a unimodal distribution of apical diameter corresponding to primarily tapered fibers (Figure 1J–L), and the mean apical diameter of tapered fibers was similar across species (Appendix S3).

The percentage of tapered fibers for each accession was considered the ground truth for comparison with the computer vision method. For the *Gh* accessions, the ground truth was 59.7%, 80.4%, or 52.5% tapered fibers for Deltapine 90, Coker 312, and Half and Half, respectively, and similar values were determined using the computer vision method (Table 2). The greatest deviation from ground truth for the *Gh* accessions was 1.5% fewer tapered fibers identified by computer vision in Half and Half.

**Table 2:** Final comparison of the percentages of tapered (T) fibers determined manually (M) or by the best machine learning (ML) algorithm in six cotton accessions. The same 24 images for each accession were annotated using the M or ML methods. The number of fibers annotated by hand was fixed at 720 in each set.

Species	Accession	M fiber number	M % T	ML fiber number	ML % T	Difference %T, M vs. ML
*G. hirsutum*	Deltapine 90	720	59.7	860	58.3	1.4
	Coker 312	720	80.4	707	79.6	0.8
	Half and Half	720	52.5	1178	54.2	1.74
*G. barbadense*	Phytogen 800	720	100	1350	97.7	2.3
	Pima 3‐79	720	100	881	98.4	1.6
	Pima S7	720	100	921	97.9	2.1

For all three *Gb* accessions, the ground truth was manually determined to be 100% tapered. However, in each case, the computer vision method identified a small percentage of *Gb* fibers as having hemisphere shape. The lowest percentage of tapered fibers detected by computer vision was 97.7% in Phytogen 800, implying that 2.3% was the greatest deviation from ground truth for the *Gb* accessions. The results for Pima S7 were similar (97.9% tapered), whereas computer vision identified 98.4% tapered fibers in Pima 3‐79 (Table 2).

In all but one accession, computer vision identified more fibers within 24 images than the 720 fibers that were manually identified to serve as the ground truth. A minimum of 707 fibers were identified by computer vision in Coker 312 and a maximum of 1350 fibers were identified in Phytogen 800 (Table 2). The size of the ovule fragments and extent of spreading of fibers as individuals versus in entangled groups can only be partially controlled during manual tissue dissection and mounting. Variations in these parameters may explain why more fibers were detected in *Gb* cv. Phytogen 800, with mainly tapered fibers, and *Gh* cv. Half and Half, with a mixture of fiber shapes, as compared with the other accessions analyzed. Overall, the detection of more fibers in an accession is anticipated to increase the accuracy of the results.

**Figure 4:**
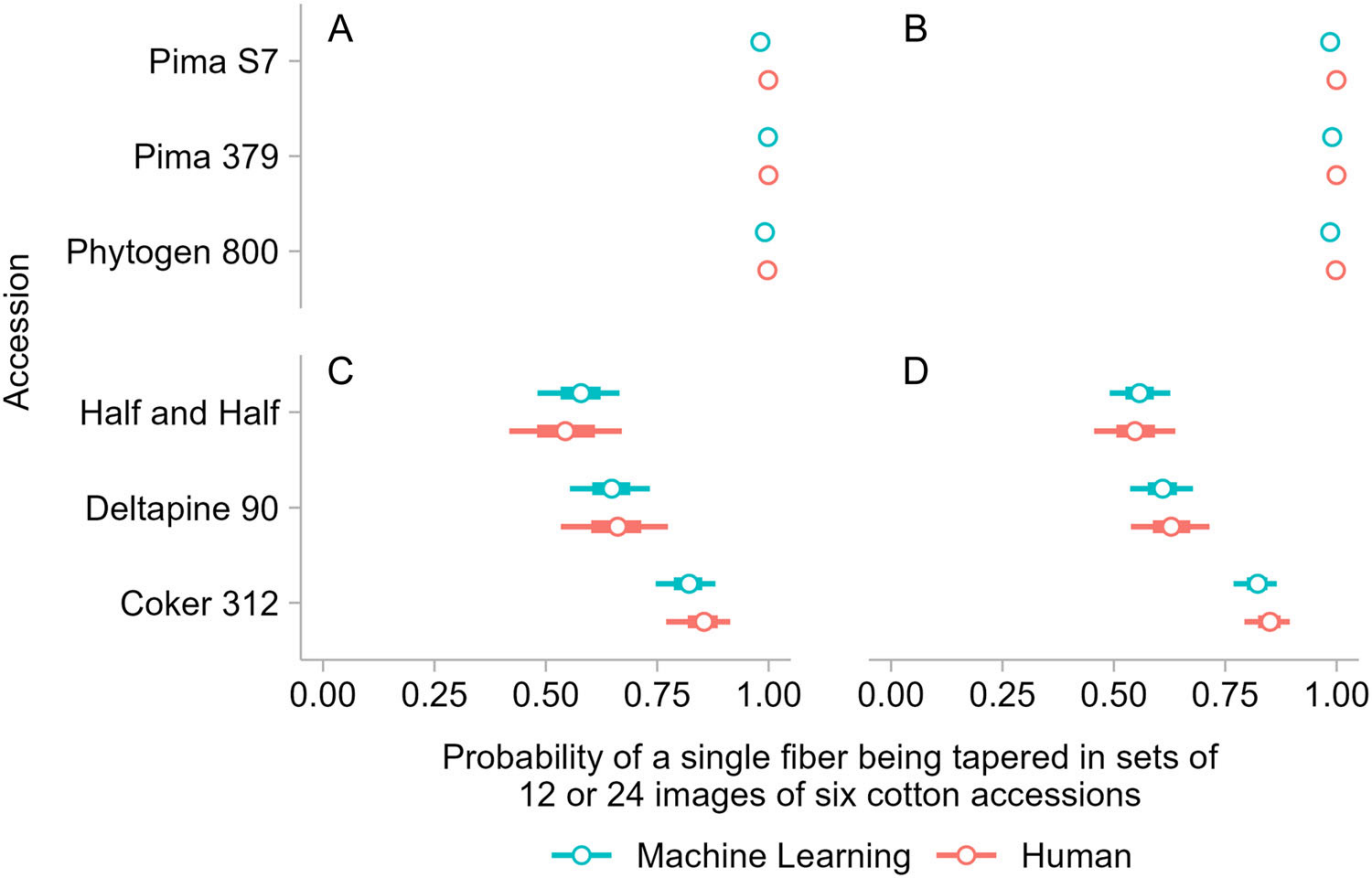
Comparison of the estimates of representation of tapered fibers after human/manual (orange) or machine learning (aqua) detection and annotation. For both methods, the point estimates and, if relevant, credible intervals (thick line is 66% and thin line is 95%) are shown, as determined by a Bayesian statistical analysis. The results for the six accessions were similar for (A, C) a random subset of 12 images and (B, D) the full set of 24 images, with increased certainty arising from more images when both fiber shapes were well represented in the *Gossypium hirsutum* (*Gh*) populations.

We considered whether 24 images were required for reliable estimates of the percentage of tapered fibers (Figure 4). The computer vision analysis of either 24 images or a random set of 12 images yielded similar point estimates for close to 100% tapered fibers in *Gb* accessions. For the three *Gh* cultivars in the evaluation set, we found similar estimates for the percentage of tapered fibers in the 12 and 24 image sets, but downsampling to 12 images substantially increased the uncertainty in the estimates (compare intervals in Figure 4). The increase in uncertainty was greater in the accessions with tapered:hemisphere ratios closer to 1:1. The binomial distribution has variance proportional to p(Tapered) × (1 – p(Tapered)). Higher variance in the fiber distribution may result in additional experimental bias given the variable representation of fiber shapes in small tissue fragments. This idea was supported by the smaller credible intervals when 24 images per accession were analyzed together. The largest differences between credible intervals for 12 versus 24 images analyzed together occurred for Half and Half and Deltapine 90, which have a more balanced representation of tapered and hemisphere fibers. By contrast, an analysis of either 12 or 24 images resulted in similar credible intervals for Coker 312, with 80.42% tapered fibers, even though the fewest fibers (707) were detected by ML for this accession (Table 2). The image‐to‐image variation was lower when images were annotated by ML versus a human, allowing for more confident estimates of tapered fiber percentages in two of three *Gh* accessions analyzed using the ML method (Figure 4).

## DISCUSSION

The results reported here demonstrate a more efficient imaging protocol and a novel ML algorithm for labeling young cotton fibers as having hemisphere or tapered shapes. We analyzed the earliest initiating and elongating fibers on the chalazal end of the ovule (Stewart, 1975) to facilitate valid comparisons between accessions. We predict that the results would be similar for fibers at the micropylar end that initiate somewhat later, once those fibers have passed through the tip‐tapering phase of development. The hemisphere or tapered shapes are readily distinguished visually as described above, and they are also supported quantitatively; as calculated from published data on *Gh* cv. Deltapine 90 (Pierce et al., 2019), there was an 85% or 185% increase in fiber diameter between the apex and 200 µm back from the apex for hemisphere or tapered fibers, respectively. The new computer vision algorithm readily detected these shape differences, allowing the rapid determination of the percentage of tapered fibers in six cotton accessions. Given the superior fineness of *Gb* (Pima) cotton with nearly 100% tapered fibers, efficiently assessing the percentage of tapered fibers in diverse *Gh* (Upland) cotton accessions is an important future research goal that will be facilitated by the improved methods reported here. Notably, the average apical diameter of tapered fibers is low (4.27–5.27 µm) and similar across the *Gh* and *Gb* species (Figure 1, Appendix S3), which supports a theoretical positive impact on fiber quality of increasing the percentage of tapered fibers in future improved *Gh* cultivars, as explained in the Introduction.

The cotton accessions analyzed in this study are well known due to their importance in cotton research and/or production over many decades. Among the *Gb* accessions, Pima 3‐79 is an inbred line that originated before 1965 in an Arizona breeding program (Smith et al., 1999). *Gb* cv. Pima S7 is an elite cultivar that was released in 1991 (Smith et al., 1999), and it was planted on 51.32% of American Pima acreage in 1996. *Gb* cv. Phytogen 800 was planted on 49.8% of American Pima acreage in 2006. The *Gh* accessions reflect nearly 100 years of Upland cotton breeding. Half and Half was developed from Mexican germplasm in the early 1900s (Bowman et al., 1996) and grown in the United States through about 1950 (Smith et al., 1999). Coker 312 was selected before 1971 from the widely successful Coker 310 (Taylor and Nugent, 1971; Smith et al., 1999), and it became the preferred material for the regeneration of somatic embryos into transformed plants (Trolinder and Goodin, 1987). Deltapine 90 was planted on 11.8% of the American Upland acreage in 1990. (Acreage data are reported at https://apps.ams.usda.gov/Cotton/AnnualCNMarketNewsReports/VarietiesPlanted/.)

Computer vision detected 97.7% tapered fibers in Phytogen 800 (Table 2), which was similar to the prior human identification of 100% tapered fibers in this accession (Graham and Haigler, 2021). Automated annotation of almost all tapered fibers in this accession occurred even though the model was trained only on the mixed fiber population of Deltapine 90. Computer vision also led to the annotation of 97.9% or 98.4% tapered fibers in the other two *Gb* accessions analyzed, whereas the human annotation gave values of 100% (Table 2). Due to the inherent error in most analytical methods, we are not able to judge whether the results of the ML or manual method are closer to reality. Computer vision avoids any unconscious human bias, but a few wide pre‐taper fibers (Graham and Haigler, 2021) might be included in an image set despite our methods being designed to avoid this. Contrary to the results obtained for *Gb*, a mixture of tapered and hemisphere fibers was observed in all three *Gh* accessions analyzed, with a maximum deviation of 1.5% in the percentage of tapered fibers as compared with the ground truth (Table 2). Interestingly, the percentage of tapered fibers varied between 51% and 80% in these three *Gh* accessions, while a bimodal distribution of apical diameters was observed in each case.

Overall, these results confirm and expand prior results for *Gh* cv. Deltapine 90 and *Gb* cv. Phytogen 800 (Graham and Haigler, 2021). The identification of almost 100% tapered fibers in two additional *Gb* accessions and a mixture of tapered and hemisphere fibers in two additional *Gh* accessions supports the broad hypothesis that wide fibers occur in modern *Gh* allotetraploid accessions, but not in modern *Gb* allotetraploid accessions. This hypothesis needs to be tested by the analysis of many more *Gh* and *Gb* cotton accessions, which is now enabled by our more efficient imaging protocol and the computer vision algorithm described here.

When TIFF images were captured with the Keyence microscope followed by use of the AI‐based protocol, about 7.25 h are required to set up the samples and obtain the percentage of tapered fibers for three accessions processed in parallel. The durations of each step were approximately: 1.5 h for the tissue dissection and preparation of three 24‐well slides; 0.75 h for the manual set‐up of the Keyence system; 4 h for operator‐free automated image capture; and 1 h for the z‐stack processing of images from 72 samples. After collecting the currently reported data, we realized that initially capturing JPEG images (about 1.4 MB file size) led to the same results in less time than when TIFF images (about 6 MB file size) were captured. In the future, capturing JPEG images will save about one hour of automated image capture time and about 15 min of z‐stack processing, resulting in 6 h required to image three accessions in parallel with 3 h being operator‐free.

The computer vision annotation of fibers in the collected images of one accession requires less than two minutes, with 707–1350 fibers detected within the 24 stacked images of each of the six accessions that we analyzed. For comparison, our former manual image capture and analysis methods (Pierce et al., 2019; Graham and Haigler, 2021) required the hand focusing of different z‐planes in several dissected ovule pieces representing one accession or treatment prior to making a 2D projection by hand using Picolay (http://www.picolay.de/). More than eight hours of continuous work generated usable images of 400–600 fibers for one accession, followed by at least two hours of additional work to label the fibers (10 h total work for one accession). In summary, the currently reported improved methods make quantifying the proportions of narrow and wide young cotton fibers up to 10‐fold more efficient if JPEG images are captured, taking into account the hands‐off time allowed by Keyence imaging when three accessions are processed in parallel.

The computer vision method also typically yielded data on more fibers (up to 1350) per accession than the fixed number of 720 fibers that were annotated by hand in the six accessions finally evaluated (Table 2). The results support the ability of the computer vision algorithm to provide reliable data on accessions with mixed fiber widths from appropriately replicated samples. In our study, computer vision detected 707 to 1350 fibers for each accession while the results were also close to ground truth. Overdispersion among the images of each accession may represent a substantial limitation at low replication, especially for accessions with more balanced tapered:hemisphere ratios. Image‐to‐image variability can be mitigated by using more images and annotating more fibers per image, which is only possible with higher‐throughput laboratory and computational methods like the ones presented here. The analysis time may also be reduced when accessions with high percentages of tapered fibers are in the evaluation set by first acquiring 12 images reflecting good biological replication. If the observed percentages of hemisphere and tapered fibers are more balanced, additional images can be acquired to generate final conclusions.

The current model does a good job in matching ML predictions to ground truth for the parameter of interest (percentage of tapered fibers), which was our main goal. In the future, we could potentially improve model performance through the additional balancing of fiber widths in the training set, as well as hyperparameter optimization. We would also like to extend the computer vision algorithm to automatically extract the cotton tip diameter. With the current algorithm, it is sometimes unclear which fiber tip is detected within a given bounding box due to overlapping fibers/fiber edges. The inclusion of the fiber tip angle in the labeling process to detect oriented bounding boxes could help in isolating the detected tip so that we can automatically extract the diameter.

The methods described can now be applied in experimental studies exploring alterations of cotton fiber shape and to high numbers of replicates and accessions of cotton growing in the field. Analyzing hundreds of cotton samples is now feasible, which will aid ongoing investigations including (a) the relationship between early fiber shape and mature cotton fiber quality, and (b) the molecular basis of two morphogenetic fates (narrow versus wide) of young *Gh* fibers in varying proportions versus the dominance of narrow fibers in *Gb* (Graham and Haigler, 2021; Yanagisawa et al., 2022). Identifying the distribution of young fiber widths early in the growing season could potentially be developed into a pre‐screen for useful accessions in cotton breeding programs aiming to improve fineness or other qualities in *Gh* cotton. Research into other elongated cells with different shape classes, for example, moss caulonema and chloronema (Rensing et al., 2020), may also benefit from adaptations of the methods developed here. In general, these more efficient methods will aid further research directed toward understanding the mechanistic control of the variable shapes of elongating single cells.

## AUTHOR CONTRIBUTIONS

Conceptualization—B.P.G., A.M.H.‐K, E.L.; Formal analysis—B.P.G., J.P., G.T.B.; Funding acquisition—A.M.H.‐K., C.H.H., E.L.; Investigations—B.P.G., J.P.; Methodology—B.P.G., J.P., G.T.B., E.L.; Software—J.P., E.L.; Validation—B.P.G., J.P., G.T.B.; Visualization—B.P.G., J.P., G.T.B., E.L.; Writing—Original draft preparation—B.P.G., J.P., C.H.H., E.L.; Writing—Review, editing—All authors. All authors approved the final version of the manuscript.

### OPEN DATA BADGE

This article has earned an Open Data badge for making publicly available the digitally‐shareable data necessary to reproduce the reported results. Upon acceptance of the article computational tools and code supporting the project analysis are available through GitHub (https://github.com/USDA-ARS-GBRU/Cotton_Fiber_Computer_Vision/) and images of six cotton accessions are available through USDA Ag Data Commons (https://data.nal.usda.gov/dataset/data-efficient-imaging-and-computer-vision-detection-two-cell-shapes-young-cotton-fibers).

## Supporting information


**Appendix S1**. Three fixatives gave similar results for the distributions of the fiber apical diameters and manual annotations of tapered and hemisphere fiber shapes in new collections of 3 DPA *Gossypium hirsutum* (*Gh*) cv. Deltapine 90 ovules with attached fibers. All fixatives were tested at 1:10 tissue:fixative ratios (1 h at room temperature). The fixatives tested were (A) 4% formaldehyde plus 0.01% glutaraldehyde in modified microtubule‐stabilizing buffer (see main text); (B) low‐toxicity, formalin‐free fixative (#A5472; MilliporeSigma, Burlington, Massachusetts, USA), which was alcohol‐based; and (C) HistoChoice (now obsolete). The alcohol in the formalin‐free fixative required us to rinse and mount the samples in buffer, which was done equivalently for all tests. For all three fixatives, the results were similar to previous observations (see text). Due to its current availability and low toxicity, the formalin‐free fixative was chosen for further work.

**Appendix S2**. Training loss versus validation loss over time during training of the final model. The loss function is used in machine learning for quantifying the amount of error during the training of a deep learning model. This function is used during optimization. Lower loss indicates better correspondence between the ground truth and model predictions. Images inset into the graph represent the model performance over time on one part of one image of the test set. The bounding boxes for ground truth and predictions are denoted by green and red, respectively. Only green ground truth boxes are shown at iteration 0, prior to training the model. After about 600 iterations, most of the green ground truth boxes are overlapped by the red predicted boxes.

**Appendix S3**. Fiber tip shapes and apical dimensions determined manually for six cotton accessions, three from *Gossypium hirsutum* (*Gh*) and three from *G. barbadense* (*Gb*).

## Data Availability

Computational tools and code supporting the project analysis are available through GitHub (https://github.com/USDA-ARS-GBRU/Cotton_Fiber_Computer_Vision/) and images of the six cotton accessions used are available through USDA Ag Data Commons (https://data.nal.usda.gov/dataset/data-efficient-imaging-and-computer-vision-detection-two-cell-shapes-young-cotton-fibers).

## References

[aps311503-bib-0001] Avci, U. , S. Pattathil , B. Singh , V. L. Brown , M. G. Hahn , and C. H. Haigler . 2013. Cotton fiber cell walls of *Gossypium hirsutum* and *Gossypium barbadense* have differences related to loosely‐bound xyloglucan. PLoS ONE 8(2): e56315. 23457548 10.1371/journal.pone.0056315 PMC3572956

[aps311503-bib-0002] Bowman, D. T. , O. L. May , and D. S. Calhoun . 1996. Genetic base of upland cotton cultivars released between 1970 and 1990. Crop Science 36(3): 577–581.

[aps311503-bib-0003] Brown, N. , P. Kumar , S. Khanal , R. Singh , N. D. Suassuna , J. McBlanchett , E. Lubbers , et al. 2020. Registration of eight upland cotton (*Gossypium hirsutum* L.) germplasm lines with *qFL‐Chr. 25*, a fiber‐length QTL introgressed from *Gossypium barbadense* . Journal of Plant Registrations 14(1): 57–63.

[aps311503-bib-0004] Bürkner, P.‐C. 2017. brms: An R package for Bayesian multilevel models using Stan. Journal of Statistical Software 80(1): v080i01.

[aps311503-bib-0005] Bürkner, P.‐C. 2018. Advanced Bayesian multilevel modeling with the R package brms. The R Journal 10(1): 395–411. 10.32614/RJ-2018-017

[aps311503-bib-0006] Carpenter, B. , A. Gelman , M. D. Hoffman , D. Lee , B. Goodrich , M. Betancourt , M. Brubaker , et al. 2017. Stan: A probabilistic programming language. Journal of Statistical Software 76(1): v076i01. 10.18637/jss.v076.i01 PMC9788645 36568334

[aps311503-bib-0007] Constable, G. , D. Llewellyn , S. A. Walford , and J. D. Clement . 2015. Cotton breeding for fiber quality improvement. *In* V. M. V. Cruz and D. A. Dierig [eds.], Industrial crops: Breeding for bioenergy and bioproducts, 191–232. Springer, New York, New York, USA.

[aps311503-bib-0008] da Silva, R. L. , N. Starliper , D. K. Bhosale , M. Taggart , R. Ranganath , T. Sarje , M. Daniele , et al. 2020. Feasibility study of water stress detection in plants using a high‐throughput low‐cost system. *In* IEEE Sensors in Rotterdam, Netherlands, 2020. Website: https://ieeexplore.ieee.org/document/9278711 [accessed 23 October 2022].

[aps311503-bib-0009] Fang, D. D. 2018. General description of cotton. *In* D. D. Fang [ed.], Cotton fiber: Physics, chemistry and biology, 1–11. Springer, Cham, Switzerland.

[aps311503-bib-0010] Gabry, J. , and T. Mahr . 2022. R package version 1.9.0. bayesplot: Plotting for Bayesian Models. Website: https://mc-stan.org/bayesplot/ [accessed 9 June 2022].

[aps311503-bib-0011] Gabry, J. , D. Simpson , A. Vehtari , M. Betancourt , and A. Gelman . 2019. Visualization in Bayesian workflow. Journal of the Royal Statistical Society: Series A 182(2): 389–402.

[aps311503-bib-0012] Graham, B. P. , and C. H. Haigler . 2021. Microtubules exert early, partial, and variable control of cotton fiber diameter. Planta 253(2): 47. 33484350 10.1007/s00425-020-03557-1

[aps311503-bib-0013] Kakani, V. , V. H. Nguyen , B. P. Kumar , H. Kim , and V. R. Pasupuleti . 2020. A critical review on computer vision and artificial intelligence in food industry. Journal of Agriculture and Food Research 2: 100033.

[aps311503-bib-0014] Kelly, B. , N. Abidi , D. Ethridge , and E. F. Hequet . 2015. Fiber to fabric. *In* D. D. Fang and R. G. Percy [eds.], Cotton, 5, 665–744. American Society of Agronomy, Madison, Wisconsin, USA.

[aps311503-bib-0015] Kim, H. J. 2018. Cotton fiber biosynthesis. *In* D. D. Fang [ed.], Cotton fiber: Physics, chemistry and biology, 133–150. Springer, Cham, Switzerland.

[aps311503-bib-0016] Krizhevsky, A. , I. Sutskever , and G. E. Hinton . 2017. ImageNet classification with deep convolutional neural networks. Communications of the ACM 60: 84–90.

[aps311503-bib-0017] Lenth, R. 2022. R package version 1.7.4‐9990003. emmeans: Estimated Marginal Means, aka Least‐Squares Means. Website: https://github.com/rvlenth/emmeans [accessed 9 June 2022].

[aps311503-bib-0018] Li, Y. , Z. Cao , H. Lu , and W. Xu . 2020a. Unsupervised domain adaptation for in‐field cotton boll status identification. Computers and Electronics in Agriculture 178: 105745.

[aps311503-bib-0019] Li, Z. , R. Guo , M. Li , Y. Chen , and G. Li . 2020b. A review of computer vision technologies for plant phenotyping. Computers and Electronics in Agriculture 176: 105672.

[aps311503-bib-0020] Maharlooei, M. , S. Sivarajan , S. G. Bajwa , J. P. Harmon , and J. Nowatzki . 2017. Detection of soybean aphids in a greenhouse using an image processing technique. Computers and Electronics in Agriculture 132: 63–70.

[aps311503-bib-0021] Nhamo, L. , R. Van Dijk , J. Magidi , D. Wiberg , and K. Tshikolomo . 2018. Improving the accuracy of remotely sensed irrigated areas using post‐classification enhancement through UAV capability. Remote Sensing 10(5): 712.

[aps311503-bib-0022] Paul, A. , S. Ghosh , A. K. Das , S. Goswami , S. Das Choudhury , and S. Sen . 2020. A review on agricultural advancement based on computer vision and machine learning. *In* J. Mandal and D. Bhattacharya [eds.], Emerging technology in modelling and graphics. Advances in intelligent systems and computing, 937: 567–581. Springer Nature, Singapore.

[aps311503-bib-0023] Pei, W. , J. Song , W. Wang , J. Ma , B. Jia , L. Wu , M. Wu , et al. 2021. Quantitative trait locus analysis and identification of candidate genes for micronaire in an interspecific backcross inbred line population of *Gossypium hirsutum* × *Gossypium barbadense* . Frontiers in Plant Science 12: 763016. 34777444 10.3389/fpls.2021.763016 PMC8579039

[aps311503-bib-0024] Pierce, E. T. , B. P. Graham , M. R. Stiff , J. A. Osborne , and C. H. Haigler . 2019. Cultures of *Gossypium barbadense* cotton ovules offer insights into the microtubule‐mediated control of fiber cell expansion. Planta 249(5): 1551–1563. 30729290 10.1007/s00425-019-03106-5

[aps311503-bib-0025] R Core Team . 2022. Version 4.2.0. R: A language and environment for statistical computing. R Foundation for Statistical Computing, Vienna, Austria. Website: https://www.R-project.org/ [accessed 9 June 2022].

[aps311503-bib-0026] Rensing, S. A. , B. Goffinet , R. Meyberg , S. Z. Wu , and M. Bezanilla . 2020. The moss *Physcomitrium* (*Physcomitrella*) *patens*: A model organism for non‐seed plants. The Plant Cell 32(5): 1361–1376. 32152187 10.1105/tpc.19.00828 PMC7203925

[aps311503-bib-0027] Sadeghi‐Tehran, P. , K. Sabermanesh , N. Virlet , and M. J. Hawkesford . 2017. Automated method to determine two critical growth stages of wheat: heading and flowering. Frontiers in Plant Science 8: 252. 28289423 10.3389/fpls.2017.00252 PMC5326764

[aps311503-bib-0028] Seagull, R. W. 1990. The effects of microtubule and microfilament disrupting agents on cytoskeletal arrays and wall deposition in developing cotton fibers. Protoplasma 159(1): 44–59.

[aps311503-bib-0029] Smith, C. W. , R. G. Cantrell , H. S. Moser , and S. R. Oakley . 1999. History of cultivar development in the United States. *In* W. C. Smith [ed.], Cotton: Origin, history, technology, and production, 99–171. John Wiley and Sons, New York, New York, USA.

[aps311503-bib-0030] Stan Development Team . 2022. R package version 2.26.11. RStan: the R interface to Stan. Website: https://mc-stan.org/ [accessed 9 June 2022].

[aps311503-bib-0031] Stewart, J. M. 1975. Fiber initiation on the cotton ovule. American Journal of Botany 62(7): 723–730.

[aps311503-bib-0032] Stiff, M. R. , and C. H. Haigler . 2016. Cotton fiber tips have diverse morphologies and show evidence of apical cell wall synthesis. Scientific Reports 6: 27883. 27301434 10.1038/srep27883 PMC4908599

[aps311503-bib-0033] Sun, S. , C. Li , A. Paterson , Y. Jiang , and J. Robertson . 2018. 3D computer vision and machine learning based technique for high throughput cotton boll mapping under field conditions. *In* 2018 ASABE Annual International Meeting. American Society of Agricultural and Biological Engineers, St. Joseph, Michigan, USA.

[aps311503-bib-0034] Sun, S. , C. Li , P. W. Chee , A. H. Paterson , Y. Jiang , R. Xu , J. S. Robinson , et al. 2020. Three‐dimensional photogrammetric mapping of cotton bolls in situ based on point cloud segmentation and clustering. ISPRS Journal of Photogrammetry and Remote Sensing 160: 195–207.

[aps311503-bib-0035] Taylor, H. , and N. Nugent . 1971. SPD Hockley County: Results of 1971 Agricultural Demonstrations. Texas State Department of Agriculture, Report No. D‐801. Texas Department of Agriculture, Austin, Texas, USA.

[aps311503-bib-0036] Tian, H. , T. Wang , Y. Liu , X. Qiao , and Y. Li . 2020. Computer vision technology in agricultural automation: A review. Information Processing in Agriculture 7(1): 1–19.

[aps311503-bib-0037] Toseef, M. , and M. J. Khan . 2018. An intelligent mobile application for diagnosis of crop diseases in Pakistan using fuzzy inference system. Computers and Electronics in Agriculture 153: 1–11.

[aps311503-bib-0038] Trolinder, N. L. , and J. R. Goodin . 1987. Somatic embryogenesis and plant regeneration in cotton *Gossypium hirsutum* L. Plant Cell Reports 6: 231–234. 24248660 10.1007/BF00268487

[aps311503-bib-0039] Tuttle, J. R. , G. Nah , M. V. Duke , D. C. Alexander , X. Guan , Q. Song , Z. J. Chen , et al. 2015. Metabolomic and transcriptomic insights into how cotton fiber transitions to secondary wall synthesis, represses lignification, and prolongs elongation. BMC Genomics 16(1): 477. 26116072 10.1186/s12864-015-1708-9 PMC4482290

[aps311503-bib-0040] Wang, F. , J. Zhang , Y. Chen , C. Zhang , J. Gong , Z. Song , J. Zhou , et al. 2020. Identification of candidate genes for key fibre‐related QTLs and derivation of favourable alleles in *Gossypium hirsutum* recombinant inbred lines with *G. barbadense* introgressions. Plant Biotechnology Journal 18(3): 707–720. 31446669 10.1111/pbi.13237 PMC7004909

[aps311503-bib-0041] Wickham, H. , M. Averick , J. Bryan , W. Chang , L. D. McGowan , R. Francois , G. Grolemund , et al. 2019. Welcome to the tidyverse. Journal of OpenSource Software 4(43): 1686.

[aps311503-bib-0042] Yanagisawa, M. , S. Keynia , S. Belteton , J. A. Turner , and D. Szymanski . 2022. A conserved cellular mechanism for cotton fibre diameter and length control. In silico Plants 4(1): diac004.

[aps311503-bib-0043] Yu, J. , Y. Hui , J. Chen , H. Yu , X. Gao , Z. Zhang , Q. Li , et al. 2021. Whole‐genome resequencing of 240 *Gossypium barbadense* accessions reveals genetic variation and genes associated with fiber strength and lint percentage. Theoretical and Applied Genetics 134: 3249–3261. 34240238 10.1007/s00122-021-03889-w

[aps311503-bib-0044] Zhai, X. , A. Kolesnikov , N. Houlsby , and L. Beyer . 2021. Scaling vision transformers. arXiv 2021 [Preprint] [posted 8 June 2021]. Available at 10.48550/arXiv.2106.04560 [accessed 23 October 2022].

[aps311503-bib-0045] Zhao, N. , W. Wang , K. Jiang , C. E. Grover , C. Cheng , Z. Pan , C. Zhao , et al. 2021. A calmodulin‐like gene (*GbCML7*) for fiber strength and yield improvement identified by resequencing core accessions of a pedigree in *Gossypium barbadense* . Frontiers in Plant Science 12: 815648. 35185964 10.3389/fpls.2021.815648 PMC8850914

