# Supplementary material for: Efficient imaging and computer vision detection of two cell shapes in young cotton fibers
Source: Appl Plant Sci. 2022 Nov 26;10(6):e11503. doi: 10.1002/aps3.11503 (PMC9742826; doi:10.1002/aps3.11503)

DOI 10.1002/aps3.11503

APPENDIX S1. Three fixatives gave similar results in terms of the distributions of the apical diameters and tapered and hemisphere fiber shapes in new collections of *Gossypium hirsutum* (*Gh*) cv. Deltapine 90 ovules and fibers at 3 DPA. All fixatives were tested at 1:10 tissue:fixative ratios (1 h at room temperature). The fixatives tested were (A) 4% formaldehyde plus 0.01% glutaraldehyde in modified microtubule-stabilizing buffer (see main text); (B) low-toxicity formalin-free fixative (#A5472; MilliporeSigma, Burlington, Massachusetts, USA), which was alcohol-based; and (C) HistoChoice (now obsolete). The alcohol in the formalin-free fixative required us to rinse and mount the samples in buffer, which was done equivalently for all tests. For all three fixatives, the results were similar to previous observations (see text). Due to its current availability and low toxicity, the formalin-free fixative was chosen for further work.

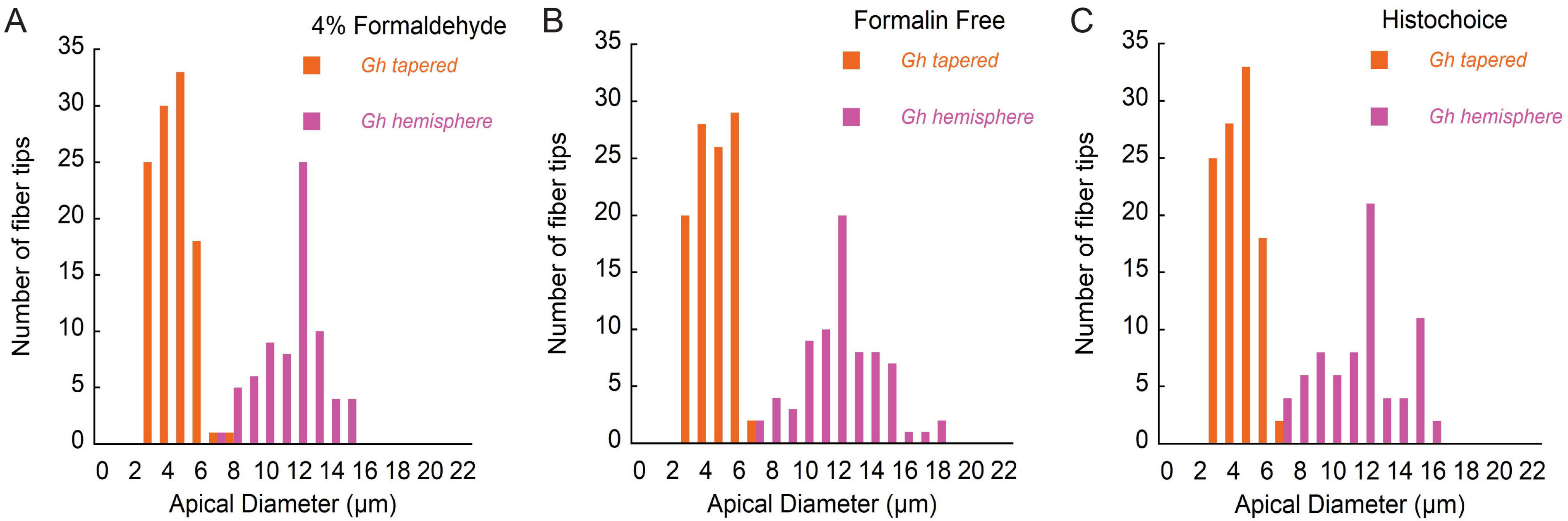

Supplement: Supplementary file 1 — Appendix S1. Three fixatives gave similar results for the distributions of the fiber apical diameters and manual annotations of tapered and hemisphere fiber shapes in new collections of 3 DPA Gossypium hirsutum (Gh) cv. Deltapine 90 ovules with attached fibers. All fixatives were tested at 1:10 tissue:fixative ratios (1 h at room temperature). The fixatives tested were (A) 4% formaldehyde plus 0.01% glutaraldehyde in modified microtubule‐stabilizing buffer (see main text); (B) low‐toxicity, formalin‐free fixative (#A5472; MilliporeSigma, Burlington, Massachusetts, USA), which was alcohol‐based; and (C) HistoChoice (now obsolete). The alcohol in the formalin‐free fixative required us to rinse and mount the samples in buffer, which was done equivalently for all tests. For all three fixatives, the results were similar to previous observations (see text). Due to its current availability and low toxicity, the formalin‐free fixative was chosen for further work. [file APS3-10-e11503-s002.pdf]
