# Supplementary material for: Efficient imaging and computer vision detection of two cell shapes in young cotton fibers
Source: Appl Plant Sci. 2022 Nov 26;10(6):e11503. doi: 10.1002/aps3.11503 (PMC9742826; doi:10.1002/aps3.11503)

DOI 10.1002/aps3.11503

APPENDIX S2. Training loss versus validation loss over time during training of the final model. The loss function is used in machine learning for quantifying the amount of error during the training of a deep learning model. This function is used during optimization. Lower loss indicates better correspondence between the ground truth and model predictions. Images inset into the graph represent the model performance over time on one part of one image of the test set. The bounding boxes for ground truth and predictions are denoted by green and red, respectively. Only green ground truth boxes are shown at iteration 0, prior to training the model. After about 600 iterations, most of the green ground truth boxes are overlapped by the red predicted boxes.

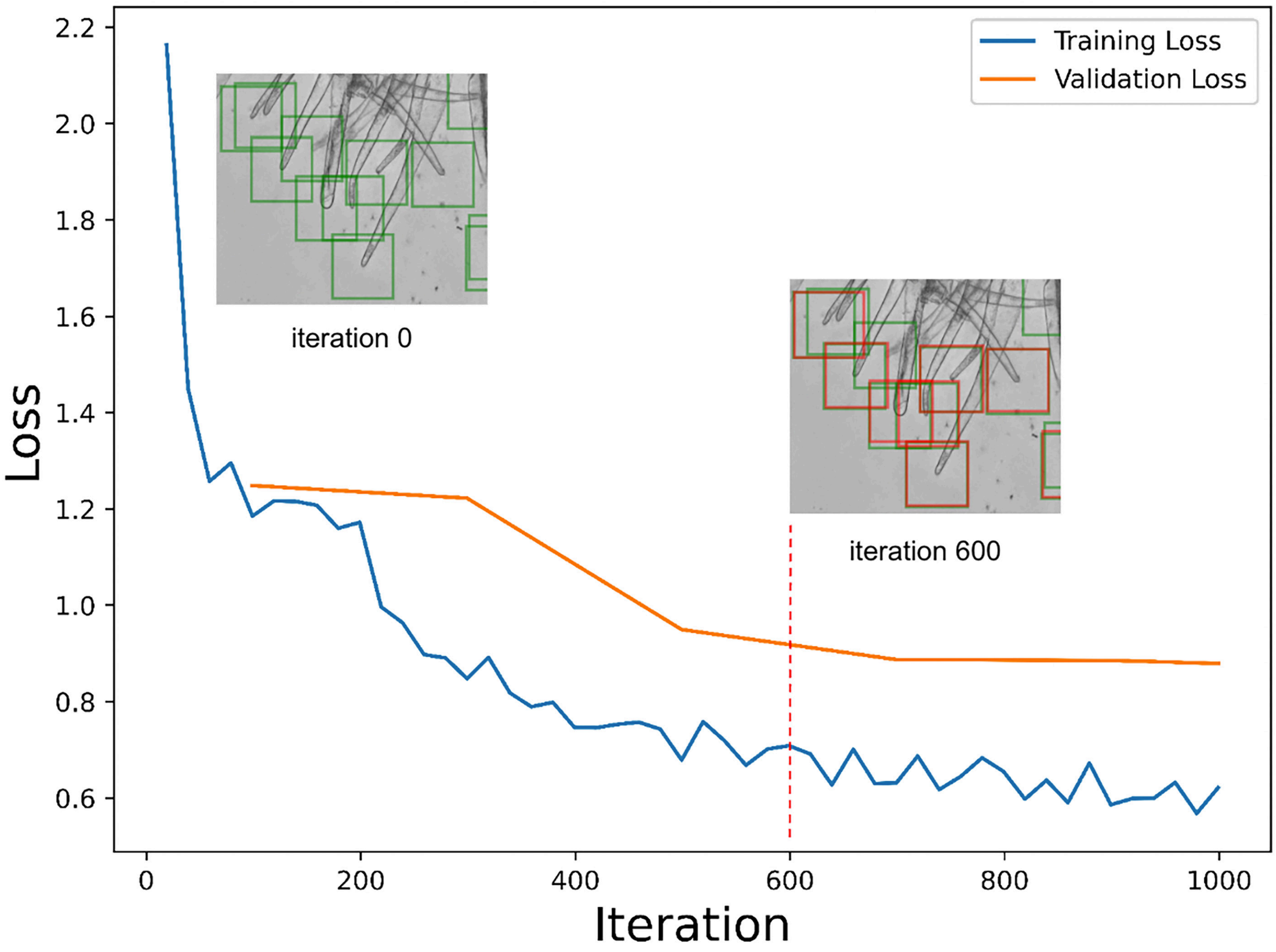

Supplement: Supplementary file 2 — Appendix S2. Training loss versus validation loss over time during training of the final model. The loss function is used in machine learning for quantifying the amount of error during the training of a deep learning model. This function is used during optimization. Lower loss indicates better correspondence between the ground truth and model predictions. Images inset into the graph represent the model performance over time on one part of one image of the test set. The bounding boxes for ground truth and predictions are denoted by green and red, respectively. Only green ground truth boxes are shown at iteration 0, prior to training the model. After about 600 iterations, most of the green ground truth boxes are overlapped by the red predicted boxes. [file APS3-10-e11503-s003.pdf]
