# Supplementary material for: Efficient imaging and computer vision detection of two cell shapes in young cotton fibers
Source: Appl Plant Sci. 2022 Nov 26;10(6):e11503. doi: 10.1002/aps3.11503 (PMC9742826; doi:10.1002/aps3.11503)
Supplement: Supplementary file 3 — Appendix S3. Fiber tip shapes and apical dimensions determined manually for six cotton accessions, three from Gossypium hirsutum (Gh) and three from G. barbadense (Gb). [file APS3-10-e11503-s001.docx]

**APPENDIX S3.**  Fiber tip types and apical dimensions determined manually for six cotton accessions, three from *Gossypium hirsutum* (*Gh*) and three from *G. barbadense* (*Gb*).

| Species | Accession | Total fibers labeled | % T  fibers | Mean apical diameter of T fibers (µm)  ± 95% CI | % H fibers | Mean  apical diameter of H fibers (µm)  ± 95% CI | Overall  mean  apical diameter  (µm)  ± 95% CI |
| --- | --- | --- | --- | --- | --- | --- | --- |
| *Gh* | Deltapine 90  (prior work) | 719^a^ | 55 | 5.53 ± 0.20 | 45 | 11.79 ± 0.30 | 8.36 ± 0.26 |
|  | Deltapine 90 | 720^b^ | 60 | 5.18 ± 0.15 | 40 | 11.81 ± 0.18 | 7.85 ± 0.26 |
|  | Half & Half | 720 | 52 | 4.86 ± 0.15 | 48 | 10.72 ± 0.15 | 7.64 ± 0.24 |
|  | Coker 312 | 720 | 80 | 5.07 ± 0.14 | 20 | 10.47 ± 0.32 | 6.13 ± 0.14 |
| *Gb* | Phytogen 800  (prior work) | 609 | 100 | 4.19 ± 0.11 | 0 | NA | 4.19 ± 0.11 |
|  | Phytogen 800 | 720 | 100 | 5.37 ± 0.07 | 0 | NA | 5.37 ± 0.07 |
|  | Pima 3-79 | 720 | 100 | 4.27 ± 0.08 | 0 | NA | 4.27 ± 0.08 |
|  | Pima S7 | 720 | 100 | 4.88 ± 0.05 | 0 | NA | 4.88 ± 0.05 |

*Note:* CI = confidence interval; H = hemisphere; NA = not applicable; T = tapered.

^a^Data from prior work on fibers of cultured ovules at four days after anthesis, previously published in: Pierce, E. T., B. P. Graham, M. R. Stiff, J. A. Osborne, and C. H. Haigler. 2019. Cultures of *Gossypium barbadense* cotton ovules offer insights into the microtubule-mediated control of fiber cell expansion. *Planta* 249: 1551–1563.

^b^All new data derive from 24 images of each accession, as described in the main text.
